# Mortality and culling of adult dairy cows in Jordan: A 3-year study (2016–2018) based on a single intensively managed dairy farm

**DOI:** 10.14202/vetworld.2022.2617-2622

**Published:** 2022-11-19

**Authors:** Zuhair Bani Ismail, Mohammad Musab Muhaffel

**Affiliations:** Department of Veterinary Clinical Sciences, Jordan University of Science and Technology, Irbid 22110, Jordan

**Keywords:** animal welfare, culling rate, dairy cows, Jordan livestock, mortality rate

## Abstract

**Background and Aim:**

Dairy cow mortality and culling are important parameters reflecting on cow health, productivity, and welfare as well as important determinants of herd sustainability, growth, and profitability. There are no published reports on the causes and rates of mortality and culling of dairy cows in Jordan. Therefore, the objectives of this study were to determine the most common causes and rates of mortality and culling of adult dairy cows in Jordan using a single well-managed dairy farm as a model over 3 years.

**Materials and Methods:**

Data extracted from the farm management record software over 3 years (January 2016–December 2018) were used in this study. Cow-specific data included the date and month of sale, death or euthanasia, age, parity, reproductive status, and daily milk yield. Cow health-specific data included physical examination findings, presumptive diagnosis, medical or surgical treatments, postmortem findings, and any available laboratory findings. Descriptive analysis was performed to determine means (± standard deviation) and frequencies of various variables using Excel Spreadsheets of Microsoft Word 10.

**Results:**

The 3-year rolling cow population in the farm used in the study was 500 ± 35. The overall mortality and culling rates were 5.9% and 28.5%, respectively. The mean age of died and culled cows was 3 ± 1.2 and 4 ± 1.5 years, respectively. The mortality rates were highest in the colder months (January through April). The most frequent causes of mortality were infectious diseases (28%), followed by non-infectious gastrointestinal diseases (25%), udder and teat diseases (mastitis 22%), and other diseases/accidents (25%). Of the infectious diseases, the most frequently diagnosed were enterotoxemia (12%), tuberculosis (TB) (8%), enteric salmonellosis (7%), and paratuberculosis (1%). The most frequently diagnosed non-infectious gastrointestinal diseases were traumatic reticulitis (11%), vagal indigestion (9%), and abomasal ulcer (5%). The most frequently diagnosed diseases causing mortality involving other body systems were reproductive diseases (acute puerperal metritis 6%), respiratory diseases (pneumonia 5% and pulmonary embolism 1%), metabolic diseases (fatty liver 3%), musculoskeletal diseases (septic arthritis 3% and downer cow syndrome 4%), neurologic diseases (unspecified causes 2%), and finally accidents (electrocution 1%). The most frequent causes of culling were old age/low milk production (39%), followed by the poor reproductive performance (31%), diseases/accidents (24%), and unidentified causes (6%). The most frequent diseases/accidents causing culling were udder diseases (mastitis 32%), followed by non-infectious gastrointestinal diseases (28%) (vagal indigestion [15%], rumen tympany [7%], and abomasal ulcer [6%]), musculoskeletal diseases (23%) (foot and claw diseases [7%], downer cow syndrome [7%], hip luxation [5%], septic arthritis [2%], and gastrocnemius rupture [2%]), respiratory diseases (pneumonia 10%), and finally infectious diseases (9%) (paratuberculosis [3%], hemorrhagic bowel syndrome [2%], and TB [2%]).

**Conclusion:**

Results of this study showed that the majority of deaths and culling of dairy cows in Jordan are due to infectious diseases followed by non-infectious gastrointestinal diseases and mastitis. More efforts aiming at improving biosecurity standards, nutritional management, and mastitis prevention measures are required to limit the impact of disease on farm economy, animal health and productivity, and animal welfare in Jordan.

## Introduction

The dairy industry in Jordan is a fraction of the overall economy, contributing to <7% of the total gross domestic product [1–3]. The entire cattle population in the country is composed of Holstein-Friesian breed, producing about 78% of the total annual market demand for fresh milk [1–4]. Unfortunately, climate change, feed and water shortages, the rising cost of feed ingredients and other farming inputs, and diseases, both infectious and non-infectious hinder significant improvements in livestock sectors in Jordan, especially dairy cattle [5, 6].

Voluntary culling of old cows and cows with reduced milk production coupled with heifer replacement programs is considered an important management tool used to ensure herd growth, sustainability, and profitability [7, 8]. Globally, diseases leading to cow death (mortality) or premature removal from the herd (involuntary culling), are considered significant constraints of dairy cattle improvement [7, 8]. In fact, recently published research has indicated an alarming rise in the annual rates of mortality and culling of dairy cows due to disease or physical injuries, raising significant concerns related to animal health and welfare among consumers and community activists [9–12].

In Jordan, there are no published reports on the causes and rates of dairy cow mortality and culling. Such important data will enable a better understanding of cow-level factors as well as farm-related management factors associated with cow health and welfare, leading to cow morbidity, mortality, and culling. Such an understanding is vital for the construction and implementation of any future national strategic plans aiming at achieving food security and sustainability by improving farm management practices, preventive animal health, and productivity of dairy cattle in Jordan.

Therefore, this study aimed to report the rates and causes of mortality and culling of adult dairy cows in Jordan using a single well-managed dairy farm as a model over 3 years.

## Materials and Methods

### Ethical approval

No ethical approvals were required in this study since no live animals were used.

### Study period and location

The study was conducted from January 1, 2016 to December 31, 2018. The total cattle population in Jordan is approximately 80,000 heads, all of which belong to the Holstein-Friesian dairy breed [1–4]. The farm used in this study is located in Al-Dhlail city. About 65% of dairy production in Jordan is produced in this region [1–4]. The average farm size ranges from 100 to 10,000 cows [1–4]. All farms share similar management and feeding practices [1–4]. Al-Dhlail area is located in North-Eastern region of Jordan between Lat. 32°07’49”N and 36°16’18”E [13]. The dry season in this region of Jordan is hot and long, spanning from May through October, while the rainy season is short, cold, and windy and spans from November through April. The estimated annual rainfall in this region is about 100 mm [13].

### Study farm

The yearly average number of cows in the farm used in this study was 500 lactating cows, 450 heifers (aged 3 months–first calving), and 420 calves aged between 1 day and 2 months (pre-weaning). Lactating cows in this farm were housed in free stall barns on dirt floors, with outside sheds available freely for the cows to use during sunny days. The cows were fed a total mixed ration composed mainly of corn silage, alfalfa hay, and various grain supplements. The average milk production in this herd ranged between 32 and 37 kg cow/day. Cows were milked 3 times/day using a 36-double herringbone milking parlor. Various hormonal-based reproductive protocols were used on the farm. The cows were bred using artificial insemination, which is routinely performed by an outside expert technician.

The lactation herd was divided into three groups according to age and stage of lactation: First-calf heifers and fresh cow group (first 21 days after calving), mature cow group, and dry cow group. The mature cow group was further divided according to daily milk yield into three subgroups: High (37−40 kg), medium (30−36 kg), and low milk production (<30 kg). The dry cow group was also divided into two subgroups: Early dry cow group (first 39 days of a 60-day dry period) and close-up cow group (last 21 days of the dry period).

During drying off, all cows were administered dry cow therapy on the 1^st^ day of the dry period using various approved drugs for this purpose. Recently, dried cows were monitored closely for 1 week. Twenty-four hours before the expected calving date, cows are moved to a clean maternity pen. Maternity pens were bedded with shredded cardboard paper. The bedding was removed between calving. The calving process was closely monitored by trained farm caretakers.

Adult cows in this farm were routinely vaccinated against foot and mouth disease, infectious bovine rhinotracheitis, bovine parainfluenza-3, bovine respiratory syncytial virus, bovine viral diarrhea, clostridial diseases, and contagious mastitis. Dry cows were also administered calfhood vaccines against *Escherichia coli*, rotavirus, and coronavirus. In addition, antiparasitic medications were administered to all cows during the dry period (ivermectin at 200 mcg/kg, SQ, or fenbendazole 10% at 5−10 mg/kg, orally).

### Farm veterinary services

Veterinary services on the farm were provided by the farm resident veterinarian 24 h, 7 days/week. Specialized veterinary consultation was provided by the principal investigator (PI) of this study (Professor Zuhair Ismail) on regular farm visits (once or twice weekly) and on emergency requests. All sick cows and cows under veterinary treatment were housed in a designated hospital pen. Lame cows and those diagnosed with infectious diseases, including contagious mastitis, were housed in separate groups and milked last. All cows destined for culling were subjected to a complete physical examination by the farm veterinarian. Animals that died naturally or were euthanized on the farm were subjected to a complete necropsy procedure. A presumptive diagnosis was made by the PI of the study based on the physical examination and postmortem findings. The cause of death or euthanasia and the reason for culling were then entered into the farm management software. All management practices, including feeding, milking, calving, neonatal calf rearing, disease diagnosis, veterinary therapeutics, and treatments, were performed on the farm according to specific standard operative protocols prepared by the PI of the study.

### Data collection

The farm management record software (DelPro; DeLaval, USA) was used to extract data related to cows sold for slaughter, died, or euthanized between January 2016 and December 2018. Individual cow-specific data included the date and month of sale, death or euthanasia, age, parity, reproductive status, and daily milk yield. Cow health-specific data included physical examination findings, medical or surgical treatments, postmortem findings, and any available laboratory findings. Cows that were sold to other farms for production purposes were excluded from the analysis.

### Statistical analysis

The collected data were entered into Excel Spreadsheets (Microsoft Word 10, Microsoft Co. USA). The data were categorized into four groups: Group 1 included dead cows (natural death plus euthanasia) due to diseases/accidents, Group 2 included cows sold for slaughter because of old age/low milk production, Group 3 included cows sold for slaughter because of poor reproductive performance, and Group 4 included all cows that were sold for slaughter, died, or euthanized and a specific reason could not be made (unidentified causes). Calculations of means (± standard deviation) and frequencies were made based on the rolling average of cows over the 3-year study period.

## Results

### Cow mortality rate and causes

**Figure-1:**
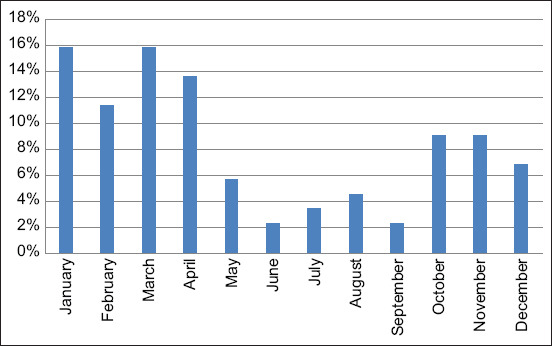
Mortality rate of adult dairy cows per month over a 3-year period (2016−2018) in a single intensively managed dairy farm in Jordan (n = 88).

The rolling 3-year (2016−2018) mean (± standard deviation) of cow numbers in the farm used in the study was 500 ± 35. The total number of dead cows over the 3-year study period was 88 with an overall yearly mortality rate of 5.9%. The mean value of age and parity of dead cows was 3 ± 1.2 years and 2 ± 0.5, respectively. The monthly mortality rate is presented in Figure-1. The mortality rates were highest over the colder months (16% in January and March, 14% in April, 11% in February, 9% in October and November, and 7% in December), while the lowest mortality rates were reported during the warmer months of the year (6% in May, 5% in August, 3% in July, and 2% in June and September).

**Figure-2:**
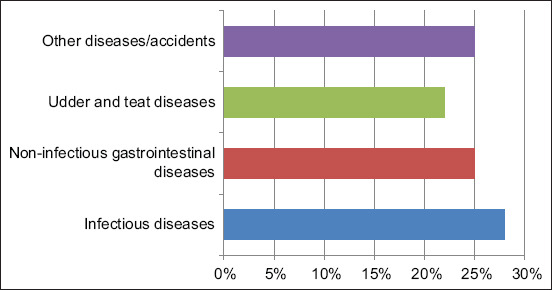
Major categories of mortality causes of adult dairy cows over a 3-year period (2016−2018) in a single intensively managed dairy farm in Jordan (n = 88).

The reported causes of cow mortality over the 3-year study period are presented in Table-1 and Figure-2. Categorically, the most frequent causes of mortality were infectious diseases (28%), followed by non-infectious gastrointestinal diseases (25%), udder and teat diseases (mastitis 22%), and other diseases/accidents (25%). Of the infectious diseases, the most frequently diagnosed were enterotoxemia (12%), tuberculosis (TB) (8%), enteric salmonellosis (7%), and paratuberculosis (1%). The most frequently diagnosed non-infectious gastrointestinal diseases were traumatic reticulitis (11%), vagal indigestion (9%), and abomasal ulcer (5%). The most frequently diagnosed diseases causing mortality involving other body systems were reproductive diseases (acute puerperal metritis 6%), respiratory diseases (pneumonia 5% and pulmonary embolism 1%), metabolic diseases (fatty liver 3%), musculoskeletal diseases (septic arthritis 3% and downer cow syndrome 4%), neurologic diseases (unspecified causes 2%), and finally accidents (electrocution 1%).

**Table-1:** Body systems and diseases/accidents causing mortality (n = 88) and culling (n = 102) of adult dairy cows over a 3-year period (2016−2018) in a single intensively managed dairy farm in Jordan.

Body system/diseases	Mortality (n = 88)		Culling (n = 102)	
	
	No.	%	No.	%
Infectious	25	28	9	9
Enterotoxemia	11	12	0	0
Tuberculosis	7	8	2	2
Enteric salmonellosis	6	7	3	3
Paratuberculosis	1	1	2	2
Hemorrhagic bowel syndrome	0	0	2	2
Gastrointestinal	22	25	28	27
Vagal indigestion	0	0	15	15
Traumatic reticulitis	10	11	0	0
Vagal indigestion	8	9	0	0
Rumen tympany	0	0	7	7
Abomasal ulcer	4	5	6	6
Udder (clinical mastitis)	19	22	32	31
Reproductive (acute puerperal metritis)	5	6	0	0
Respiratory	5	6	10	10
Pneumonia	4	5	10	10
Pulmonary embolism	1	1	0	0
Metabolic (fatty liver)	3	3	0	0
Musculoskeletal	6	7	23	23
Hip luxation	0	0	5	5
Foot and claw diseases	0	0	7	7
Septic arthritis	3	3	2	2
Downer cow syndrome	3	4	7	7
Gastrocnemius rupture	0	0	2	2
Neurologic (unspecified causes)	2	2	0	0
Accidents (electrocution)	1	1	0	0

### Culling rate and causes

**Figure-3:**
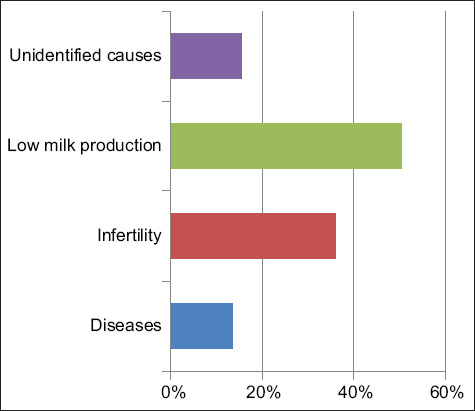
Major categories of culling causes of adult dairy cows over a 3-year period (2016−2018) in a single intensively managed dairy farm in Jordan (n = 427).

The total number of cows culled during the 3-year study period was 427, with an overall yearly cull rate of 28.5%. The mean age and parity of culled cows during this period were 4 ± 1.5 years and 3 ± 1, respectively. The percentages of culled cows according to reason are presented in Figure-3. Categorically, the most frequent causes of culling were old age/low milk production (39%), followed by the poor reproductive performance (31%), diseases/accidents (24%), and unidentified causes (6%).

**Figure-4:**
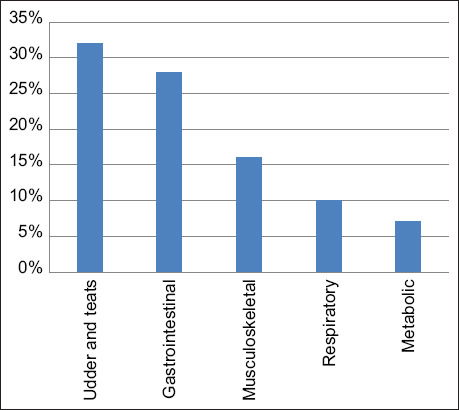
Body systems involved in diseases causing culling of adult dairy cows over a 3-year period (2016−2018) in a single intensively managed dairy farm in Jordan (n = 102).

The percentages of cows culled because of diseases/accidents over the 3-year study period are presented in Table-1 and Figure-4. The most frequent diseases/accidents causing culling were udder diseases (mastitis 32%), followed by non-infectious gastrointestinal diseases (28%) [vagal indigestion (15%), rumen tympany (7%), and abomasal ulcer (6%)], musculoskeletal diseases (23%) [foot and claw diseases (7%), downer cow syndrome (7%), hip luxation (5%), septic arthritis (2%), and gastrocnemius rupture (2%)], respiratory diseases (pneumonia 10%), and finally infectious diseases (9%) [paratuberculosis (3%), hemorrhagic bowel syndrome (2%), and TB (2%)].

## Discussion

In this study, the most frequent causes and overall rates of mortality and culling of adult dairy cows in Jordan are reported for the 1^st^ time. The average ages of dead and culled cows in this study were 3 ± 1.2 years and 4 ± 1.5 years, respectively. The total lifespan of Holstein dairy cattle from birth to death has been reported to range between 4.5 and 6 years [14]. However, the productive life of the cow does not start until the first calving, which typically occurs at 2 years of age. More than 80% of milk is produced by the cow during the next 2.5–4 years [14]. Although, the results of this study showed that culled cows are removed from the production herd at a slightly older age (4 ± 1.5 years) compared to dead cows (3 ± 1.2 years), these results indicate that many dairy cows in Jordan could leave the production herd at an earlier age compared to the natural expected lifespan of dairy cows (4.5−6 years) [14]. This constitutes a significant loss in milk production and raises serious concerns over cow’s health, management standards, and potential animal welfare status.

In this study, the overall mortality rate was 5.9%. This mortality rate is just slightly above the acceptable mortality rate (1−5%) of dairy cows kept under intensive conditions [7, 12]. A literature search regarding the causes of mortality and culling of dairy cows typically reveals large variations among countries, regions, and individual farms with large proportions of unknown or unidentified causes [15]. These variations are attributed to differences in disease definition or classification, differences in local and farm conditions related to management, feeding, housing, and disease control practices [15]. In this study, it was possible to identify the specific cause of death in most cases because of the implementation of strict protocols related to disease diagnosis, treatment, and necropsy. The low death rate in this study also indicates that sick cows are promptly treated on the farm. This may also indicate that in developed countries, where death rates are typically higher, sick cows are sold for slaughter or euthanized sooner because of higher welfare standards [16].

In this study, the mortality rates were highest over the colder months (16% in January and March, 14% in April, 11% in February, 9% in October and November, and 7% in December) while the lowest mortality rates were reported during the warmer months of the year (6% in May, 5% in August, 3% in July, and 2% in June and September). The climate in Northeast Jordan, where the study farm is located is characterized by an average ambient temperature ranging from 41°F (5°C) during the colder months and 92°F (33°C) during the summer [13]. In dairy cattle, the suggested comfortable neutral temperature ranges from –5 to 25°C [17, 18]. In general, higher cow death rates are reported during the hottest seasons due to increased heat index [19]. In our study, the most likely explanation for why more cows reportedly died during the colder months is poor housing conditions. Cows on this farm are housed in free-stall barns with outside lots and sheds available; during the rainy season, the floors become muddy and extremely unhygienic, leading to increased cow morbidity and mortality. Another factor could be wind and extreme temperature fluctuations between day and night during winter months, leading to increased mortality in this study. It has been suggested that factors related to barn microenvironments such as temperature, relative humidity, and air velocity, are associated with increased cow mortality during winter months [20, 21].

The overall yearly culling rate in this study was 28.5%. These results are similar to the reported culling rates in other countries, such as the Netherlands (25.4%), Northwest Spain (26%), the UK (30%), and Canada (28.2) [12]. Lower (21.3% in Ireland) and higher culling rates (40% in Australia and 36% in USA) have also been reported [12]. These large variations in the culling rates between countries are most likely due to differences in the criteria used to select cows for culling, in addition to variations in production and management systems [12].

In this study, the major causes of culling were old age/low milk production (39%), poor reproductive performance (31%), and diseases/injuries (24%). These results are similar to previously published data which indicated that reproductive diseases followed by low milk production and mammary gland diseases were the most prevalent causes of culling of dairy cows in Spain [12].

In this study, diseases/accidents (including infectious and non-infectious diseases) were the major causes of cow mortality and culling. This is alarming and constitutes a significant concern over severe economic losses and cow welfare. This mortality and culling are involuntary and management goals are typically set to reduce the yearly proportion of cows eliminated because of these causes. Infectious diseases (28%), including enterotoxemia, bovine TB, enteric salmonellosis, and paratuberculosis were the most frequent causes of cow death in this study. Enterotoxemia is a fatal hemorrhagic enteritis caused by various strains of *Clostridium perfringens*. Although the disease is more common in young growing animals, adult cattle are still susceptible, especially during periods of environmental stress. Enterotoxemia has been associated with a sudden change in diet or situations where nutritional management is inconsistent, leading to drastic disturbances in the intestinal microenvironment and rapid growth of the intestinal bacteria and production of powerful toxins [22]. In addition to routine vaccination against clostridial diseases, effective control requires reducing environmental stress by optimizing housing conditions as well as improving the nutritional management of the herd. Bovine TB was the second most common cause of cow death in this study. Bovine TB is considered a significant zoonotic and endemic disease in Jordan, leading to significant annual economic losses [23]. Effective control of this disease requires the implementation of vigorous test and cull policy, strict biosecurity measures, and reduction of cow density to reduce the environmental burden of bacteria [24]. Enteritis caused by *Salmonella* spp. and *Mycobacterium avium* paratuberculosis (Johne’s disease) were common causes of death in cows in this study. The previous report has indicated that the prevalence rates of *Salmonella* spp. and Johne’s disease in dairy farms in Jordan were 23% and 65%, respectively [25]. Control of both diseases requires the implementation of strict biosecurity measures and improved housing conditions.

The second most category of diseases causing cow mortality in this study was non-infectious gastrointestinal diseases (25%). These included traumatic reticulitis, vagal indigestion, and abomasal ulcers. Traumatic reticulitis is a potentially fatal disease leading to acute peritonitis, chronic peritonitis, and ultimately vagal indigestion due to restrictions on gastrointestinal motility or fatal pericarditis. Although, there are no reported cases of traumatic reticulitis in dairy cows in Jordan, clinical evidence is overwhelming. Adequate prevention of this fatal condition is routine administration of oral magnets at an early age and removal of sharp metallic objects from the environment. Abomasal ulcers are another important disease commonly diagnosed in dairy cows around parturition due to stress, hormonal, and nutritional factors [26]. Worldwide, the estimated prevalence of abomasal ulcers in cows ranges between 11 and 49% [27]. Prevention requires careful environmental and nutritional management of the dairy farm. Udder and teat diseases (mastitis 22%) were the third most common causes of cow mortality in this study. These results are in agreement with previously published data [21]. Mastitis is prevalent in farms with poor environmental hygiene and improper milking machine function and milking routine. Prevention of mastitis can be achieved by improving stall hygiene, ensuring the proper function of the milking machine, and standardization of milking practices.

## Conclusion

The overall mortality and culling rates of adult dairy cows in this study were 5.9% and 28.5%, respectively. The major causes of mortality were infectious diseases, non-infectious gastrointestinal diseases, and clinical mastitis, while the major causes of culling were low milk production, poor reproductive performance, and diseases/accidents. These results indicate that more efforts aiming to improve dairy cow nutritional management and infectious disease and mastitis prevention, including biosecurity, vaccination, and standardizing milking routine, are required to limit the impact of disease on animal health, productivity, and welfare in Jordan.

## Authors’ Contributions

ZBI: Designed the study, analyzed the data, and prepared the manuscript. MMM: Collected the data. Both authors have read and approved the final manuscript.
